# *Ralstonia solanacearum* and *R. pseudosolanacearum* on *Eucalyptus*: Opportunists or Primary Pathogens?

**DOI:** 10.3389/fpls.2017.00761

**Published:** 2017-05-11

**Authors:** Teresa A. Coutinho, Michael J. Wingfield

**Affiliations:** Department of Microbiology and Plant Pathology, Forestry and Agricultural Biotechnology Institute, University of PretoriaPretoria, South Africa

**Keywords:** bacterial wilt, abiotic stress, biotic stress, opportunism, clonal *Eucalyptus*

## Abstract

*Ralstonia solanacearum* and *R. pseudosolanacearum* are well known primary pathogens of herbaceous crops. Reports of wilt caused by these pathogens in tree species are limited other than on *Eucalyptus* species. Despite the widespread occurrence of so-called bacterial wilt on eucalypts in tropical and sub-tropical parts of Africa, Asia, and the Americas, there remain many contradictions relating to the disease. Our field observations over many years in most regions where the disease occurs on *Eucalyptus* show that it is always associated with trees that have been subjected to severe stress. The disease is typically diagnosed by immersing cut stems in water and observing bacterial streaming, but the identity of the bacteria within this suspension is seldom considered. To add to the confusion, pathogenicity tests on susceptible species or clones are rarely successful. When they do work, they are on small plants in greenhouse trials. It has become all to easy to attribute *Eucalyptus* death exclusively to *Ralstonia* infection. Our data strongly suggest that *Ralstonia* species and probably other bacteria are latent colonists commonly occurring in healthy and particularly clonally propagated eucalypts. The onset of stress factors provide the bacteria with an opportunity to develop. We believe that the resulting stress weakens the defense systems of the trees allowing *Ralstonia* and bacterial endophytes to proliferate. Overall our research suggests that *R. solanacearum* and *R. pseudosolanacearum* are not primary pathogens of *Eucalyptus*. Short of clear evidence that they are primary pathogens of *Eucalyptus* it is inappropriate to attribute this disease solely to infection by *Ralstonia* species.

## Introduction

Bacterial wilt is one of the world’s most destructive plant diseases. Two of the causal agents, *Ralstonia solanacearum* and *R. pseudosolanacearum,* have a broad host range infecting plants in over 50 families (Hayward, 2000), with reports of new hosts appearing regularly (Denny, 2006). The majority of hosts are herbaceous plants. In these pathosystems the causal agent/s is/are considered to be the primary pathogen. In the case of woody hosts (Supriadi et al., 2001), the pathosystem is much less well understood.

Based on phylogenetic evidence, the two *Ralstonia* species causing bacterial wilt of *Eucalyptus* appear to have evolved along two primary lines of descent: one in the Americas (*R. solanacearum*) and the other (*R. pseudosolanacearum*) having both an Asian and African origin (Prior and Fegan, 2005; Carstensen et al., 2017). It is thus likely that the movement of *Eucalyptus* from its primary center of origin in Australia to many countries worldwide, has led to these trees being exposed to local populations of these two bacterial species. This is consistent with the fact that these bacteria are known to be “highly flexible” and capable of rapid adaptation to new hosts and environmental changes (Peeters et al., 2013).

Bacterial wilt was first reported in *Eucalyptus* plantations in China (Cao, 1982) and Brazil (Sudo et al., 1983), the centers of diversity of the *Ralstonia* spp. Other than a “new host record” (Akiew and Tremorrow, 1994) in Australia where most *Eucalyptus* species are native, outbreaks of bacterial wilt in native stands of *Eucalyptus* or in established plantations, have rarely been reported (Old et al., 2003). Bacterial wilt became problematic only when *Eucalyptus* species were established in intensively managed plantations and the problem worsened when the industry moved toward clonal propagation from cuttings (Alfenas et al., 2006, 2009; Wang et al., 2014).

Our research over the past 15 years in most areas of the tropics and substropics where bacterial wilt has been recorded, suggests that *R. solanacearum* and *R. pseudosolanacearum* are not primary pathogens of *Eucalyptus*. All our evidence suggests that the *Eucalyptus* disease and death associated with these bacteria is closely linked to stressful conditions arising from both biotic and abiotic factors. In this review, we present evidence supporting this hypothesis.

## Symptoms

The causal agents of bacterial wilt are soilborne and infection generally occurs through the roots (Hayward, 1991), either at the site of lateral root emergence or through wounds. After entry into the host tissue they rapidly multiply, invade the root cortex and vascular parenchyma cells intercellularly, before spreading to the xylem vessels (Vasse et al., 1995). In these vessels they degrade the xylem wall components, parenchyma cells and pit membranes, resulting in masses of bacteria and cellular debris (Agrios, 2005). Thereafter they spread to the aerial parts of the host eventually blocking the water transport system.

The age, health and nutritional status of the host, environmental conditions and aggressiveness of the pathogen will determine the speed and severity of wilt symptom development (Hayward, 1991). In the nursery, external symptoms of bacterial wilt in *Eucalyptus* cuttings and seedlings include darkening of the stem base, leaf and stem necrosis, wilting and root death. When latently infected plants are transplanted to the field, they usually begin to show symptoms 4 months after planting (Alfenas et al., 2006). However, when the temperatures exceed 30°C, symptoms are expressed within 1–2 months after planting (Wardlaw et al., 2010).

**FIGURE 1:**
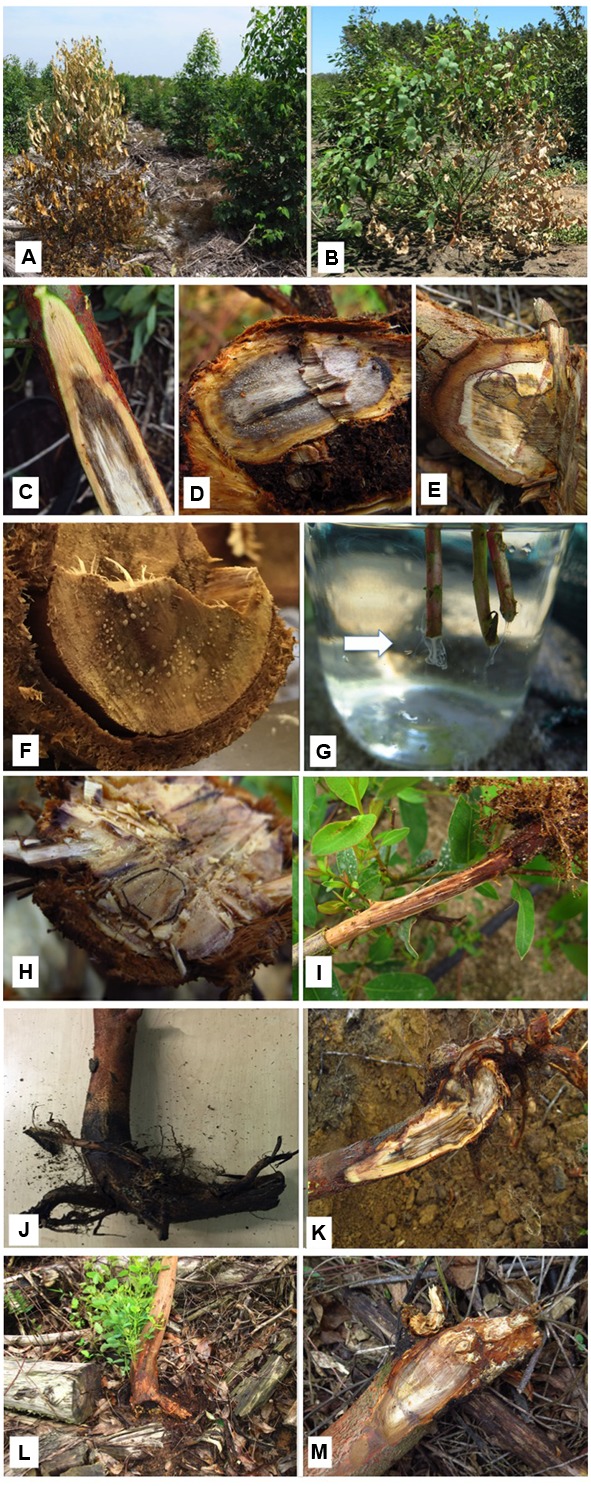
**Symptoms of bacterial wilt on eucalypts. (A)** Entire tree showing signs of having died rapidly with leaves retained. **(B)** Infected tree commonly display partial death with one or a few branches wilted. This is linked to occlusion of parts but not all of the vascular tissue. **(C)** Internal discolouration of the vascular tissue of an infected tree. **(D)** Internal discolouration of the vascular tissue in a severely infected tree showing (exudation of bacteria. **(E)** Section of the base of a partially infected tree showing wood discoloration associated with an impacted root. **(F)** Bacterial exudateevident on the cut surface of an infected tree. **(G)** Bacteria seen exuding from the freshly cut stems (arrow) of infected nursery plants placed in water. **(H)** Section through the base of an asymptomatic tree but where smallareas of bacterial exudate are present. These trees with effective root systems typically recover without symptom development. **(I)** Poorly developed root system of an infected tree which would not be able to sustain itsgrowth. **(J)** J-rooting arising from poor planting practice typically associated with bacterial infection. **(K)** Section through the base of a tree showing compacted roots and discoloration associated with bacterial infection. **(L)** Epicormic shoots commonly develop at the bases of trees withknotted roots and that are also infected with bacteria. Stress associated with these poor root systems allows bacteria to proliferate. **(M)** Base of a young tree infected with bacteria but also with the root rot pathogen, *Ganoderma philippii*. *Ralstonia pseudosolanacearum* is commonly found in tree suffering fromthis root rot disease.)

Trees between 2 and 4 years of age have been found to be the most susceptible to infection by *Ralstonia* species (Wardlaw et al., 2010). The most common external symptoms in these diseased *Eucalyptus* trees are the reddening and wilting of the foliage (**Figure 1A**) and leaf drop. Occasionally only branches or sections of the crown show signs of wilt (**Figure 1B**). Internal symptoms include vascular discolouration of the wood (**Figures 1C–E**). When the stem is sectioned, bacterial exudate is seen either exuding from the cut surface (**Figure 1F**) or if placed in water, exuding from the plant tissue (**Figure 1G**).

The most common approach to identifying bacterial wilt in *Eucalyptus* planting operations is to observe bacterial exudation from cut surfaces. Foresters do this routinely, and laboratory-level identification of the bacteria is seldom pursued. If bacterial exudation is observed from the plant material, the immediate assumption is rapidly made that a *Ralstonia* species is solely responsible for tree death. While convenient, we have found this conclusion to be seriously misleading. Our laboratory analyses, based on culture independent and dependent methods, have shown that other bacterial species are often present in the exudate (Carstensen, unpublished), but their role in the observed wilt disease is unknown. Clearly there is a need for greater rigor in making diagnoses.

## Susceptibility of *Eucalyptus*

*Eucalyptus* species differ in their susceptibility to infection by *Ralstonia* species. For example, Old et al. (2003) considered *Eucalyptus pellita* and *E. urophylla* to be amongst the most susceptible species. However, this is probably linked to the fact that these two species are most commonly planted in the tropics and sub-tropics where *Ralstonia* species are best adapted to infect these plants. Variation in susceptibility in vegetatively propagated clones of interspecific hybrids is well known. Over the past 50 years, the bulk of the research on this disease on *Eucalyptus* has focused on screening for resistant/tolerant material (Gan et al., 2004; Li et al., 2007; Wang et al., 2011; Marques et al., 2013; Fonseca et al., 2016). Identifying this material and deploying it in plantations is an obvious solution to managing bacterial wilt. However, the screening process is fraught with difficulties.

An essential requirement for a screening program is a reliable and reproducible inoculation method. In the case of *Ralstonia* species, artificial inoculations on *Eucalyptus* are rarely successful, i.e., inoculations either fail or inconsistent results are obtained (Cruz and Dianese, 1986; Dianese et al., 1990; Dianese and Dristig, 1993; Coutinho et al., 2000; Wei et al., 2014). Fonseca et al. (2016) described what they termed an “efficient method” to test for resistance of *Eucalyptus* to *R. solanacearum* but when attempted elsewhere (Carstensen et al., 2017), the method failed to produce symptoms in a susceptible clone. The common failure to prove Koch’s postulates for *Ralstonia* species on *Eucalyptus* provides an indication that there are factors, other than the susceptible host and an aggressive pathogen, that play a role in this pathosystem. Interestingly, in a study on ironwood (*Casuarina equisetifolia*), the majority of seedlings inoculated with *R. pseudosolanacearum* shown to be associated with wilt in the field, also failed to produce symptoms in a greenhouse (Ayin et al., 2015). This phenomenon may thus be a common feature of a woody host-*Ralstonia* species interaction.

## Disease Occurrence

The lifestyle of *Ralstonia* species further supports our hypothesis that they are opportunists. They are similar to other “environmental pathogens,” i.e., those bacteria able to thrive in the environment in the absence of a host (Morris et al., 2009), and a term used to describe some opportunists in phytobacteriology (Takikawa, 2012). As saprophytes they can survive for many years in soil (Graham and Lloyd, 1979) and in water courses (Elphinstone, 2005). They are reportedly capable of surviving in these environments in a viable but non-culturable form (Xu et al., 1982), a mechanism used to overcome adverse environmental conditions (Grey and Steck, 2001). When a susceptible host is planted in infested fields, the pathogen infects the plants through the roots and thereafter colonizes the host tissue. In Vietnam, eastern Thailand and Indonesia, for example, it has been observed that when *E. camaldulensis* was planted in fields previously occupied by cassava, a host of this bacterium (Machmud, 1986), the incidence of bacterial wilt increased (Old et al., 2003).

*Ralstonia* species can colonize some hosts asymptomatically as latent infections (Hayward, 1991), but this phenomenon in the *Ralstonia* pathosystem is poorly understood. Host stems may contain large bacterial populations but are asymptomatic (Swanson et al., 2007). Latently infected propagative material of, for example, potato, banana, and geranium, is believed to be the major route for long distance dispersal of these pathogens (Buddenhagen, 1986; Janse, 1996; Janse et al., 2004). In the case of *Eucalyptus* moving apparently healthy plants from the nursery to the field has led to disease outbreaks (Mafia et al., 2012; Thongsen et al., 2013). Once planted in the field, we believe the resulting transplant stress can trigger symptom expression.

A question has arisen as to whether or not *R. solanacearum* and *R. pseudosolanacearum* can reside as asymptomatic infections within *Eucalyptus* tissue. We have found these bacteria in the roots of vigorously growing trees (**Figure 1H**). This is often in the root systems at the point where cuttings were originally made and where small areas of necrotic tissue is found. It is clear that in these cases, the bacteria are not associated with disease. In plant bacteriology the term “endophyte” is restricted to those intercellular bacteria that impart an ecological benefit to the plant without causing any harm (Bacon and Hinton, 2007) and thus excludes latent pathogens. However, Hayward (1991) observed that *Ralstonia* species have the ability to multiply asymptomatically in numerous plant species (non-hosts), and designated these plants as “distant hosts.” The xylem vessels are not invaded in these hosts, and the bacteria either occur in the root cortex or on the root surface (Alvarez et al., 2008). They are considered reservoirs of the bacteria and include numerous weed species (Hayward, 1991; Wenneker et al., 1999). In the wider context of plant pathology, they could be considered as endophytes or epiphytes. Studies to identify bacterial endophytes in *Eucalyptus* have been undertaken using both culture dependent and independent methods (Ferreira et al., 2008; Procópio et al., 2009; Esposito-Polesi et al., 2015), but *R. solanacearum sensu lato* was not detected. *Eucalyptus* is thus not a distant host of these pathogens and plants could become infected if the conditions are favorable for this to occur.

*Eucalyptus* trees are colonized by a range of both fungal (Slippers and Wingfield, 2007) and bacterial (Ferreira et al., 2008; Procópio et al., 2009; Esposito-Polesi et al., 2015) endophytes. In the case of fungi, some are considered latent pathogens, e.g., members of the Botryosphaeriaceae, and when the tree is stressed by either biotic and/or abiotic factors, disease symptoms develop (Slippers and Wingfield, 2007). We are of the opinion that invasion and colonization of the host by *Ralstonia* spp. and subsequent blockage of the water flow (the plant is stressed), initiates infection by these endophytic fungi. Death of the tree then results from a complex of biotic and abiotic stress factors. This situation would be similar to that observed in the *Erwinia psidii/Eucalyptus* interaction (Coutinho et al., 2011) where infection by the bacterium lead to further branch/tree dieback due to species in the Botryosphaeriaceae.

## Stress Factors Associated with Infection

Unlike the situation in the herbaceous plant-*Ralstonia* pathosystems, stress factors appear to be important in predisposing *Eucalyptus* trees to infection by *R. solanacearum* and *R. pseudosolanacearum*. These factors include malformed root systems (j-rooting) as a result of poor planting practices, deployment of overgrown nursery plants (**Figure 1I**), physiological problems, e.g., boron deficiency (Bernard and Xu, 2006), injuries to the root collar caused by excessive temperatures (Mariano et al., 2013) and typhoon damage (Zhou et al., 2008). It is unclear whether these stress factors merely allow the bacteria entry into the plants through the creation of wounds, or whether they weaken the host’s defense systems allowing these pathogens already present in the plant tissues to colonize the internal host tissue.

Our observations over many years have shown that one of the most common factors associated with bacterial wilt in *Eucalyptus* is root knotting (**Figures 1I,J**). This is a character that can be associated with particular clones that do not develop effective root systems (**Figure 1K**), which is genetically determined. It can, however, also manifest when clones are planted in highly compacted or dense soils. The result in both cases is that trees are under extreme stress. They tend to grow actively for about 6 months after which they die. Epicormic shoots will also develop at the base of infected trees (**Figure 1L**). It is thus common to see large numbers of trees appearing to die very rapidly. In this case, they have clearly reached a point where the knotted root systems cannot sustain the upper parts of the trees; a condition where *Ralstonia* species are able to multiple rapidly to block the xylem systems. In this regard, it is known that water stress, due to a blocked vascular system, leads to the over production of defense compounds (pectins and tyloses) which reduces the resistance of the host to infection (Kannan and Bastas, 2015).

The presence of other pathogens may also result in plant stress. For example, an important pathogen of *Eucalyptus* species in South East Asia is *Ganoderma philippii* (Coetzee et al., 2011). Roots with advanced infections caused by this fungal pathogen are commonly co-infected with *R. pseudosolanacearum* (**Figure 1M**), which illustrates the opportunistic nature of *Ralstonia* species. Stress placed on the tree by the primary pathogen (*G. philippii*) could result in opportunistic invasion of this bacterial species. Interestingly, when isolations are made from the exudate produced in trees infected with *Ralstonia* species, other bacterial species are commonly isolated. Fungi are rarely isolated, even if general isolation media are used. The bacterial genera commonly isolated include *Ralstonia* (most abundant bacterium), *Clostridium, Bacillus, Enterobacter, Klebsiella,* and *Pseudomonas* (Carstensen, unpublished) With the exception of *Bacillus* (Paz et al., 2012), none of the other genera have been reported to occur in healthy *Eucalyptus* species as endophytes. All five genera have, however, been isolated separately and together from numerous tree species with wetwood or slime flux symptoms (Sinclair and Lyon, 2005). There have been no reports of this “disease” in *Eucalyptus*. However, at advanced stages of infection by *Ralstonia* species, the wood is discolored and has a “wet” appearance.

## Primary Pathogen or an Opportunist?

When outbreaks of bacterial wilt are reported in *Eucalyptus* plantations infected trees are typically scattered throughout the affected area. Rarely are all trees infected, even where the plantations have been established with a single, susceptible clone. When the root systems of diseased trees are excavated, in every case that we have examined and this includes plantations in countries of Africa, Asia, and South America, they are either knotted, malformed or they have been infected by well-known primary root pathogens. We believe that these stressful situations have weaked the defense systems of the trees, allowing *Ralstonia* species already present in the trees to rapidly invade and block the vascular tissues of the host. In some cases, it appears that other bacteria, probably also endophytic colonists of the trees, contribute to the occlusion of the vascular system. Trees with healthy, well-developed root systems in the same plantations are rarely affected even when they might be infected with *Ralstonia* species. If trees do show a low level of disease symptoms, we have observed them recover and this has also been reported by Coutinho et al. (2000) and Old et al. (2003).

The definition of an opportunistic plant pathogenic bacterium, as adapted from the medical literature characterizes them as pathogens that infect plant hosts that are predisposed to infection due to abiotic and/or biotic (other than the primary pathogen) stress factors (Coutinho, unpublished). In this case we would then accept that in the case of the *Ralstonia/Eucalyptus* pathosystem, the cause of tree death is not due to a single, primary agent, but rather to a complex suite of factors. Plants respond to different stresses and stress combinations in a highly complicated manner. Thus the illustration that abiotic factors in combination with *Ralstonia* occurs in this pathosystem, will require investigations into the changes that occur in the host at the cellular and the physiological as well as at the transcriptome levels.

## Conclusion

While *Ralstonia* species are the primary cause of bacterial wilt in herbaceous crops, most available evidence suggests that is not true in the case of their association with *Eucalyptus*. Abiotic stress factors, usually linked to poor root systems, inappropriate planting practices, poor site factors or infections by primary pathogens such as *G. philippii* appear to be necessary for infection to occur. Under such stressful conditions the *Ralstonia* species apparently often present in planting stock from nurseries, are able to multiply rapidly and to block the vascular tissues. In some cases, other biotic agents, such as fungal endophytes may also contribute to tree death. Our evidence and much of the literature suggests strongly that so-called bacterial wilt observed in *Eucalyptus* plantations is due to a complex of both biotic and abiotic agents and not solely due to *Ralstonia* infections.

Trees reported to be dying of bacterial wilt typically do so in the first few months after planting. Most commonly, the planting stock has been produced by vegetative propagation of selected clones. There is clear evidence that some clones are more susceptible than others. Our experience is that the susceptible clones are those that have poor root systems (a common problem with some clones) or root systems that do not develop actively on stressful sites. *Eucalyptus* clonal nurseries are chronically infected with *Ralstonia* species and apparently healthy plants leaving these nurseries are commonly infected with these bacterial pathogens. Where these plants experience stressful conditions after planting, they commonly display symptoms of bacterial wilt. Those that “capture” the site, i.e., have a well developed root system, remain healthy or they recover after showing mild symptoms. This is despite the fact that the majority of them are infected with bacteria.

When *Eucalyptus* trees display rapid wilt in plantations, a common response by foresters is to look for bacterial exudate. Where this is seen, the immediate response is that the trees are suffering from a biotic disease problem. This provides a convenient diagnosis that in most cases paints an incomplete picture of the real situation. No effort is made to examine the root systems of the trees or to seek other possible causes of tree death. From an alternative standpoint, resolving stress factors such as those relating to the genetics of the selected tree crop or site factors is more complex than attributing the problem to a biotic factor. Simply attributing tree death to *Ralstonia* infection based on bacterial exudate is a “red herring” and diagnoses based solely on this observation should be avoided if long term plantation sustainability is expected.

## Author Contributions

TC and MW wrote the article. MW provided the majority of the photographs.

## Conflict of Interest Statement

The authors declare that the research was conducted in the absence of any commercial or financial relationships that could be construed as a potential conflict of interest.
